# Loop-Mediated Isothermal Amplification-Based Workflow for the Detection and Serotyping of *Salmonella* spp. in Environmental Poultry Flock Samples

**DOI:** 10.3390/foods13244069

**Published:** 2024-12-17

**Authors:** Patricia Regal, Anne Doval, Iria García-Ramos, Alberto Cepeda, Alejandro Garrido-Maestu, Alexandre Lamas

**Affiliations:** 1 Food Hygiene, Inspection and Control Laboratory (LHICA-USC), Department of Analytical Chemistry, Nutrition and Bromatology, Faculty of Veterinary Science, Campus Terra, Universidade de Santiago de Compostela (USC), 27002 Lugo, Spain; patricia.regal@usc.es (P.R.); anne.doval@rai.usc.es (A.D.); iria.garcia.ramos@rai.usc.es (I.G.-R.); alberto.cepeda@usc.es (A.C.); 2 Laboratory of Microbiology and Technology of Marine Products (MicroTEC), Instituto de Investigaciones Marinas (IIM—CSIC), Eduardo Cabello, 6, 36208 Vigo, Spain

**Keywords:** loop-mediated isothermal amplification, *Salmonella* spp., *S.* Typhimurium, *S*. Enteritidis, *S*. Infantis, *S*. Hadar, *S*. Virchow, poultry, environment, farm

## Abstract

*Salmonella* spp. is one of the most important foodborne pathogens worldwide. Given the fact that poultry and poultry products are the main source of human infection, Salmonella control in these farms is of utmost importance. To better control this pathogen in farms, boot swabs are used to sample farm environments but the analysis of these swabs is mainly based on culture-dependent methods. In the present study, a novel loop-mediated isothermal amplification (LAMP) method was developed for the rapid screening of *Salmonella* spp. in boot swab samples from broiler flock environments. Four different DNA extraction protocols were evaluated in depth, including a simple thermal lysis, a chelex-based protocol and two thermal lysis protocols followed by the purification of magnetic beads made of silica (“glass milk”) in order to determine the most suitable alternative for potential on-site, farm analyses. The methodology evaluation included a blind interlaboratory assay and as a proof-of-concept, a naked-eye colorimetric assay was also included. Following the final methodology, it was possible to reach an LoD_50_ of 1.8 CFU/25 g of the samples, with a high relative sensitivity (95.7%), specificity (100%) and accuracy (96.6%) along with Cohen’s kappa of concordance with respect to the ISO standard 6579-1:2017 of 0.9, with an RLOD of 1.3. In addition to this, due to the relevance of certain serotypes with the genus *Salmonella* spp., a serotype LAMP panel for the specific identification of *S.* Typhimurium, *S.* Enteritidis, *S.* Infantis, *S.* Hadar and *S.* Virchow was also developed. Even though some degree of cross-reactivity among the primers developed was observed, all the serotypes could be accurately identified based on their melt curve analysis profile. Taken together, in the present study, a rapid *Salmonella* spp. screening method, suitable for farm applications, was developed, along with a serotyping panel that could be used in a laboratory setup for the identification of the most relevant serotypes of the genus, taking advantage of real-time amplification followed by melt curve analysis.

## 1. Introduction

The poultry market, through the production of meat and eggs, is one of the most important sectors worldwide due to its influence in global markets [1]. Additionally, this industry plays a crucial role in maintaining food safety. Poultry products are one of the main sources of *Salmonella* spp. in the food chain. The control of this pathogen is carried out from primary production to retail markets. Based on the European Union Regulation (EC) No 2160/2003 [2], member states have developed their national plans for *Salmonella* control in poultry production. These plans include environmental self-monitoring sampling by the operator to determine the presence of *Salmonella* spp. in farms. One sampling method, exclusively used in the case of broilers, involves walking inside the breeding facility with boot swabs, which are later analyzed for the presence of *Salmonella* spp. [3]. This procedure has been reported to be the most sensitive and cost-effective approach for the detection of *Salmonella* spp. in farms [4,5]. In addition to the genus, certain serotypes are considered particularly relevant for human health, namely *S*. Typhimurium, *S*. Enteritidis, *S*. Infantis, *S*. Virchow, and *S*. Hadar [6].

The ISO 6579-1:2017 is the reference method for *Salmonella* detection in the food chain [7]. This standard applies to food and environmental control in the food industry, as well as environmental samples from primary production. The method is based on classical microbiology, involving two enrichment steps, selective media seeding, and confirmation through biochemical methods. A minimum of three days is needed to classify a sample as negative and a minimum of five days to determine if a sample is positive. These turnaround times are further extended due to serotyping using antisera. This delays the production system, and may pose a challenge for an industry operating on narrow economic margins.

Alternative methods for the detection of *Salmonella* spp., such as immunoassays like the VIDAS or molecular methods, have been developed in recent decades [8,9,10]. Molecular methods, particularly real-time PCR (qPCR), have gained importance in pathogen detection in the food chain as they allowed us to overcome the limitations of classical, culture-based methods [11,12]. While these methods still require a prior enrichment step, the times are significantly shortened, and results can be obtained within 24 h. Although qPCR is the gold standard among molecular amplification methods, it has some limitations, primarily requiring relatively complex equipment capable of temperature ramping and fluorescence detection [13,14].

In recent years, a series of molecular isothermal amplification techniques, such as Recombinase Polymerase Amplification (RPA) or loop-mediated isothermal amplification (LAMP), have emerged [15,16]. In these techniques, amplification occurs at a constant temperature in a simple thermal block or water bath. Moreover, these techniques support the introduction of chemicals that enable visual detection without the need for fluorescence measurement [17,18].

The current study presented two main goals. The first was to develop a LAMP-based method to detect the presence of *Salmonella* in environmental samples from poultry farms in less than 24 h, along with a proof of concept of a low-cost, colorimetric format for future laboratory assay decentralization. The second goal consisted of a subsequent step to be performed on *Salmonella*-positive samples, and was focused on a panel of independent LAMP assays for rapid identification of the presence of some of the most relevant serotypes for the poultry industry, namely *S*. Typhimurium, *S*. Enteritidis, *S*. Infantis, *S*. Virchow, and *S*. Hadar.

## 2. Materials and Methods

### 2.1. Bacterial Strains and Culture Preparation

All bacterial strains used in the present work are listed in Table 1. Specifically, *Salmonella* Enteritidis WDCM 00030 was selected as the reference strain for the development and optimization of the LAMP assays for the detection of *Salmonella* spp., as well as the *S.* Enteritidis serotype-specific LAMP. *S.* Virchow LHICA C11/22, *S.* Infantis LHICA C12/20, *S.* Hadar LHICA C11/21, and *S.* Typhimurium WDCM 00031 were used to develop the corresponding serotype-specific LAMP assays. Additional *Salmonella* serotypes, as well as non-*Salmonella* bacterial species, were used for inclusivity/exclusivity assays. All strains were conserved in cryovials at −20 °C. The preparation of fresh cultures was performed by transferring one cryoball to a 25 mL flask with 10 mL of Brain Heart Infusion (BHI, Merck Millipore, Burlington, MA, USA) and incubated with agitation (150 rpm) at 37 °C during 18 h. Upon completion, the strains were plated in nutrient agar (NA, VWR, Barcelona, Spain) and incubated at 37 °C for 24 h. Finally, the plates were conserved and 4 °C until use. For spiking assays, *Salmonella* strains were grown as described and the culture was serially diluted and samples inoculated with different volumes of different dilutions. Also, dilutions were plated in NA to determine bacterial concentration. Buffered Peptone Water (BPW, Merck Millipore, Darmstadt, Germany) was used for sample enrichment. Semisolid Rappaport Vassiliadis medium (MRVS, DifcoTM, BD, Madrid, Spain) was used for *Salmonella* selective enrichment. Xylose Lysine Deoxycholate agar (XLD, Thermo Scientific, Oxoid) and RAPID’*Salmonella* agar (Bio-Rad, Hercules, CA, USA) were used for *Salmonella* isolation. Media were used according to ISO 6579-1:2017 [19].

### 2.2. Primer Design

For the detection of *S.* Virchow, *S.* Hadar, *S.* Infantis and *Salmonella* spp., new sets of primers were designed with Primer Explorer V5 (https://primerexplorer.jp/e/index.html, 16 December 2024). For the detection of *Salmonella* spp., the primers reported by Costa-Ribeiro et al., targeting the *ttr* gene, were selected [20], while for the serotypes Typhimuriums and Enteritidis, the primers described by Azinheiro et al. were selected and targeted the genes STM4497 and *safA*, respectively [21]. In Table 2, a complete list of all the primers used in the present study is provided.

For the design of the new sets of primers, the reference genomes retrieved from RefSeq were used, including NZ_CP025094.1 (group_27174), NZ_CP121068.1 (group_21126), and NZ_CP022069.2 (group_29846) for *S.* Virchow, *S.* Hadar, and *S.* Infantis, respectively.

### 2.3. Nucleic Acid Extraction

In the present work, four different DNA extraction methods were evaluated from simple thermal lysis to other methods that include different types of purification. In all cases, 1 mL of enriched sample was transferred to a 1.5 mL microtube and centrifuged at 900× *g* for 1 min to eliminate sample debris (step omitted for pure bacterial culture). The supernatant was transferred to a new microtube and centrifuged at 16,000× *g* for 1 min. The supernatant was discarded, and the pellet was used for DNA isolation. All the DNA samples were conserved at −20 °C until use.

#### 2.3.1. Thermal Lysis (TL)

The pellet was resuspended in 100 µL of nuclease-free water and heated at 99 °C for 5 min at 1400 rpm in a heater block (Thermomixer, Eppendorf AG, Wesseling-Berzdorf, Germany). Then, the sample was centrifuged again at 16,000× *g* for 1 min to eliminate bacterial and sample debris and the supernatant was transferred to a new microtube.

#### 2.3.2. Chelex

The pellet was resuspended in 100 µL of 6% Chelex^®^100 (Bio-Rad Laboratories, Inc., Hercules, CA, USA). The sample was incubated for 15 min at 56 °C and 1400 rpm, and then heated at 99 °C for 8 min at 1400 rpm. Then, the samples were centrifuged at 16,000× *g* for 1 min and the supernatant was transferred to a new tube.

#### 2.3.3. Thermal Lysis with Magnetic Bead Purification

DNA was isolated as described in Section 2.3.1, but, in this case, the final supernatant was mixed with 100 µL of magnetic beads (Mag-Bind^®^ TotalPure NGS, Omega-Biotek, Norcross, GA, USA). The mixture was incubated for 5 min at room temperature. The beads were recovered with a magnetic particle concentrator (Dynal^®^ MPC, Invitrogen, Carlsbad, CA, USA) until the liquid was clear. The supernatant was removed, and the pellet was washed two times with 200 µL of 70% ethanol. Finally, the pellet was air-dried to eliminate the rest of the ethanol leaving the caps open and the tubes in the magnetic rack. The microtubes were retrieved from the magnetic rack and the magnetic pellet was resuspended in 100 µL of nuclease-free water. Sample was incubated for 2 min at room temperature and then the tubes were placed again in the magnetic rack. When the liquid containing the released DNA was clear, it was transferred to a new microtube.

#### 2.3.4. Thermal Lysis with “Glass Milk” Purification (GM)

This DNA extraction was based on the method described by Page, Robert, et al. with some modifications. In this case, the pellet was resuspended in 100 µL of nuclease-free water and 100 µL of a 4% SDS solution and incubated for 5 min at 99 °C and 1400 rpm in a heater block. Then, 400 µL of 100% isopropanol, 200 µL of 1.25 M NaCl and 10 µL of “glass milk” were added and the sample was incubated for 5 min at room temperature. After that, it was centrifuged for 15 s in minicentrifuge at 2000× *g*. The supernatant was discarded, and the pellet was washed two times with ethanol at 70%. Then, the pellet was air dried in a heater block at 65 °C for 5 min with lead. After that, the pellet was resuspended in 100 µL nuclease-free water to release the DNA from the silica. The sample was centrifuged at 2000× *g* and the supernatant was transferred to a new tube.

### 2.4. DNA Concentration and Quality

The comparison among the four DNA extraction protocols was performed by quantifying the DNA concentration in all the samples spiked with *Salmonella*. The quantification was performed with the dsDNA Broad Range (BR) assay kit (Invitrogen™, ThermoFisher Scientific, Waltham, MA, USA) in combination with the commercial fluorometer Qubit (Invitrogen™, ThermoFisher Scientific, Waltham, MA, USA) and DNA purity was determined with NanoDrop Lite Plus (Thermo Scientific, Waltham, MA, USA).

### 2.5. ttr-LAMP

#### 2.5.1. Real-Time *ttr*-LAMP

Fluorescent LAMP assays were performed in a QuantStudio 12k Flex Real Time PCR system (Applied Biosystems, ThermoFisher Scientific, Waltham, MA, USA). Primers were designed by targeting the *ttr* gene. Reactions were carried using 12 µL Fast Master Mix (ISO-004, OptiGene, UK), 800 nM of FIP/BIP primers, 400 nM of LB/LF primers and 200 nM of F3/B3 primers, 50 nM of CXR Reference Dye (Promega, Madison, WI, USA) and 2 μL of template DNA and the remaining volume was completed with nuclease-free water. Technical duplicates were performed for all samples, and the experiments were run at 65 °C for 30 min, with fluorescence acquisition every 30 s. Then, melt curve analysis was performed as follows: samples were heated at 95 °C for 1 s, 80 °C for 20 s, and heated up to 95 °C with temperature increments of 0.05 °C/s and fluorescence acquisition after each temperature increment. Only samples with both positive technical replicates and Tm values falling within the calculated average temperature ± its standard deviation were considered positive.

#### 2.5.2. Colorimetric *ttr*-LAMP

Colorimetric LAMP assays were performed in 1.5 mL microtubes in a heater block (Thermomixer) at 65 °C for 30 min. The reaction composition and volume were the same as the fluorescent LAMP but without ROX. After filling 1.5 mL microtubes, the top of the tube was covered with Parafilm^®^ leaving an opening of 1–2 mm on the hinge side of the tube. First, 1 μL of SYBR Green I 1000X (Invitrogen^™^, ThermoFisher Scientific, Waltham, MA, USA) was deposited in the center of Parafilm^®^. Then, the lid was carefully closed, and the tubes were incubated. After that, tubes were shaken to mix the sample with SYBR Green I and centrifuged for 10 s in a minicentrifuge (mySPIN 6, ThermoScientific, Waltham, MA, USA). Positive samples were greenish while negative samples remained orange. Furthermore, under UV light, positive samples emitted fluorescence, while the negative ones did not.

#### 2.5.3. Serotyping LAMP

Once the presence of *Salmonella* was confirmed, i.e., *ttr*-LAMP positive, the serotyping LAMP was applied. A real-time, fluorescence-based LAMP to determine the five serotypes of importance in poultry production (*S.* Typhimurium, *S.* Enteritidis, *S.* Infantis, *S* Virchow, *S.* Hadar) was developed. The list of LAMP primers designed for this purpose are included in Table 2. The reaction conditions were the same as that of the *ttr*-LAMP. The acceptance criteria were the same defined as those for the *ttr*-LAMP in Section 2.5.1.

### 2.6. LAMP Validation

#### 2.6.1. Evaluation of the Inclusivity and Exclusivity

For inclusivity assays, 54 *Salmonella* strains belonging to 31 different serotypes and 3 different subspecies were included. For exclusivity assays, strains from 28 other bacterial strains, belonging to 18 different species, were included. In Table 1, a detailed list of the microorganisms included in the present study is provided. Pure cultures were prepared as described in Section 2.1, and the DNA of this pure culture was extracted with the method described in Section 2.3.1.

#### 2.6.2. Dynamic Range

The dynamic ranges covered with the different protocols described in Section 2.2 were evaluated with pure DNA extracted from the strain WDCM 00030, as well as with a feces-spiked sample. After preparing the pure culture, and the spiked sample, the DNA was extracted with all the DNA extraction protocols, it was quantified, ten-fold serially diluted in nuclease-free water (Promega, Madison, WI, USA) and analyzed in technical triplicates by *ttr*-LAMP.

Regarding the chicken feces, 100 µL of *Salmonella* overnight pure culture was diluted in 900 µL of feces in BPW. DNA was isolated with the four methods described in Section 2.2. Then, isolated DNA was serially diluted as for the pure culture.

#### 2.6.3. Determination of the Limit of Detection (LoD) and Relative Limit of Detection (RLOD)

The Limit of Detection (LoD) with confidence of 50% (LoD_50_) and 95% (LoD_95_) was determined for the four DNA extraction methods tested as described by Wilrich and Wilrich [22]. Regarding the Relative Limit of Detection (RLOD), the model described by Mărgăritescu and Wilrich was used [23,24]. To determine these limits, chicken bedding was collected from a chicken farm and the absence of *Salmonella* was determined by ISO 6579-1:2017. Two pairs of boot swabs were placed in a stomacher bag with 25 g of chicken bedding collected and spiked with different concentrations of *Salmonella* Enteritidis WDCM 00030. Then, samples were homogenized with 225 mL of pre-warmed BPW and incubated for 18 h at 37 °C. After that, 1 mL of the sample was collected and processed as indicated in Section 2.2 for DNA isolation with the four different methods tested. In addition, the samples were analyzed by the reference method ISO 6579-1:2017. Briefly, after BPW incubation, 0.1 mL was transferred to modified MRVS. Plates were incubated at 41.5 °C for 48 h. Suspected samples were streaked in XLD and RAPID’ *Salmonella* plates and incubated for 24 h at 37 °C. Samples with presumptive *Salmonella* colonies were confirmed with the latex agglutination test (Microgen Bioproducts Ltd., Surrey, UK).

#### 2.6.4. Fitness-for-Purpose

Once the LoD_50_ with each different DNA extraction method was determined, all the samples above the corresponding value were considered and classified as being in Positive or Negative Agreement (PA/NA) if the *ttr*-LAMP result matched that obtained by the ISO reference method and were considered to be Positive or Negative Deviations (PD/ND) if the results did not match the reference method. Once classified, these values were used to determine the relative sensitivity, specificity and accuracy (SE, SP and AC, respectively) along with Cohen’s kappa of concordance (k) as previously described by Anderson et al. and Tomás et al. [8,25].

The developed LAMP method was also tested with interlaboratory tests carried out annually by the Central Veterinary Laboratory in Spain. This test includes ten samples of chicken feces inoculated with 100 CFU, 10 CFU or not inoculated. The samples were analyzed following the protocol previously described and evaluating the four DNA isolation methods described. The final workflow was also used to analyze the routine samples analyzed in the laboratory as part of the national *Salmonella* control plan.

### 2.7. Graphical Representation and Statistical Analysis

The statistical analyses and the representation of the data obtained in the present study were performed with Graphpad Prism 10 (Boston, MA, USA). One-way ANOVA analysis with Dunn’s test was used to determine the existence of differences between groups (*p* < 0.05).

## 3. Results

### 3.1. LAMP Assay Evaluation

#### 3.1.1. Inclusivity/Exclusivity

The evaluation of the inclusivity indicated that all the 31 serotypes and 54 strains tested in the present study reported positive results for the *ttr* gene with an average melting temperature (Tm) of 88.94 ± 0.18 °C. Regarding the evaluation of the exclusivity, a panel of 28 strains covering 18 different species typically encountered in food and environmental samples were tested. None of the exclusivity panel strains reported positive results, thus demonstrating the specificity of the assay.

**Figure 1:**
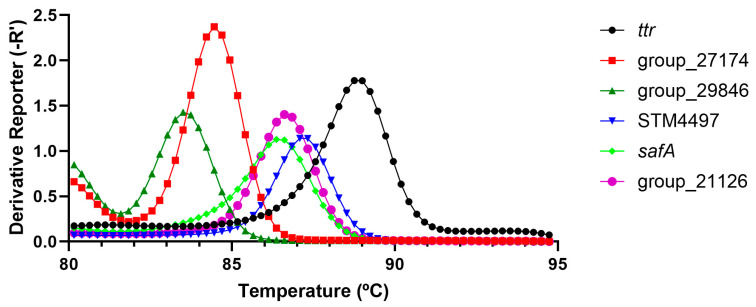
Graphical summary of the typical Tm values obtained for the *ttr* and serotyping LAMP assays. Each assay was run in simplex format.

When focusing on the serotype-specific assays, it was possible to amplify the different serotypes when using the corresponding serotype-specific LAMP assay. In this sense, the STM4497 (Typhimurium) gene reported an average Tm value of 86.98 ± 0.28 °C (10 strains including monophasic variants), *safA* (Enteritidis) reported values of 86.19 ± 0.37 °C (6 strains), and for group_29846 (Hadar, 3 strains), group_21126 (Infantis, 2 strains) and group_27174 (Virchow, 3 strains), the Tm values were 83.46 ± 0.35 °C, 86.64 ± 0.21 °C and 84.08 ± 0.28 °C, respectively, as shown in Figure 1. When focusing on the exclusivity, 20 non-target strains were tested covering 16 different serotypes, which were all positive for *ttr* but all negative for the serotype-specific LAMP assays (note that all assays were intended to be run in simplex format to avoid Tm misidentification).

#### 3.1.2. DNA Extraction Protocol Comparison and *ttr*-LAMP Dynamic Range

**Figure 2:**
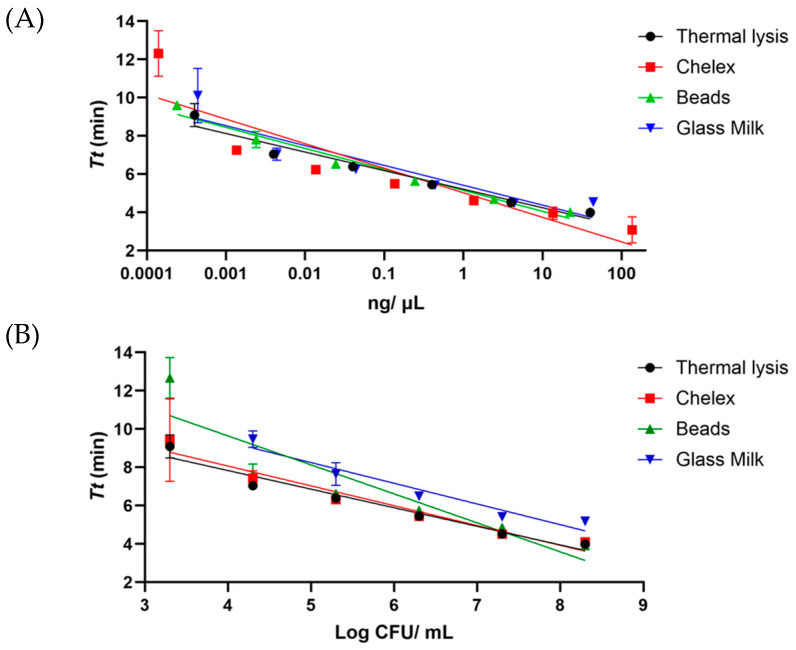
Dynamic range covered with the different DNA extraction protocols with the *ttr*-LAMP assay with pure DNA (**A**) and with bacteria inoculated in boot swabs (**B**). The amplification time is provided as *Tt*, Time to Threshold.

The dynamic range of LAMP assay was determined in pure DNA and feces, inoculated with *S.* Enteritidis WDCM 00030. In both cases, the DNA was isolated with the four methods described. When analyzing the pure bacterial DNA, all four protocols reached the range of the picograms. In this sense, with TL and GM, the lowest concentration was 0.4 pg/µL, while with the beads, the value slightly decreased down to 0.2 pg/µL and with chelex, a value of 0.1 pg/µL was reached, as shown in Figure 2A. In the case of chicken feces inoculated with *Salmonella*, differences were observed among the different DNA extraction methods. It was determined that the sample was spiked with 8.3 log CFU of *Salmonella*. Three protocols, namely chelex, TL and magnetic beads, were able to carry out detection until 3.3 log CFU/mL, while the GM method carried out detection until 4.3 log CFU/mL, as shown in Figure 2B.

### 3.2. DNA Extraction Protocol Comparison

**Figure 3:**
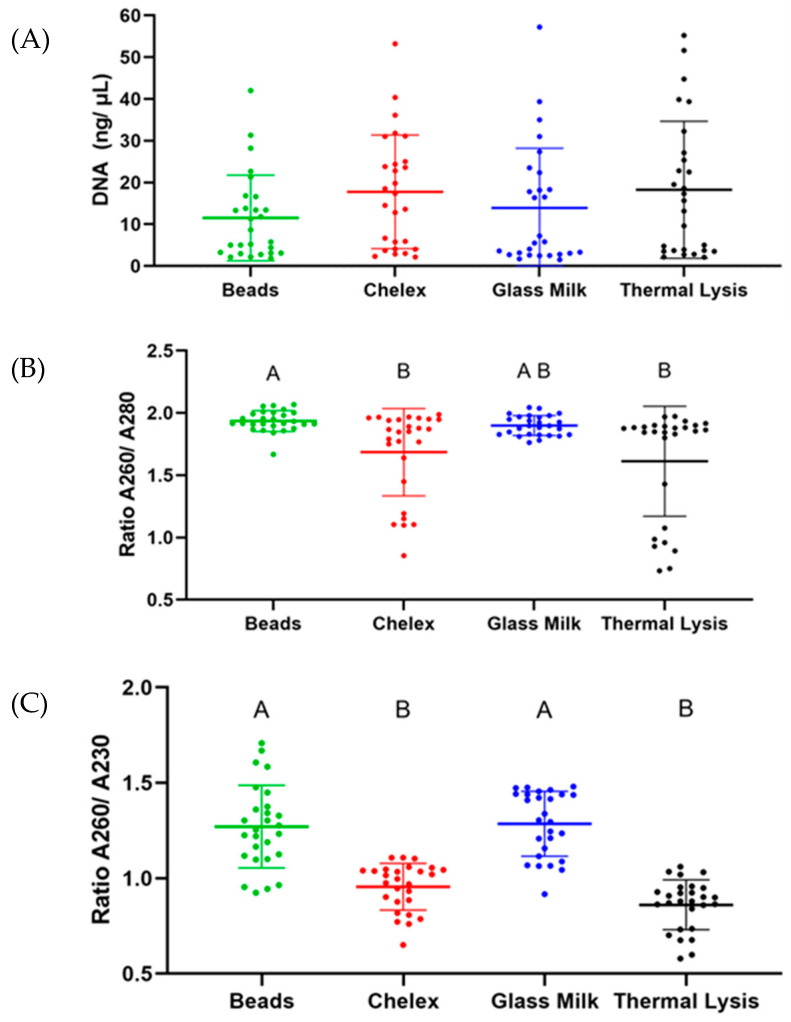
Results obtained with the different DNA extraction protocols attending to their DNA concentration (**A**), 260/280 quality ratio (**B**) and 260/230 quality ratio (**C**).

In order to better determine the performance of each extraction protocol, spiked samples were used. There were no significant differences in the quantity of DNA isolated between the different extraction protocols tested (see Figure 3A). Contrary to the DNA concentration, when the purity of the extracts was measured, significant differences were observed, i.e., the A260/A280 ratio of magnetic beads (1.935 ± 0.084) and GM (1.899 ± 0.080) was significantly higher (*p* < 0.05) than the ratio of chelex (1.685 ± 0.351) and TL (1.612 ± 0.441). The same results were observed with the ratio A260/A230, as shown in Figure 3B,C.

### 3.3. Validation of the LAMP

#### 3.3.1. Determination of the LoD and RLOD

**Figure 4:**
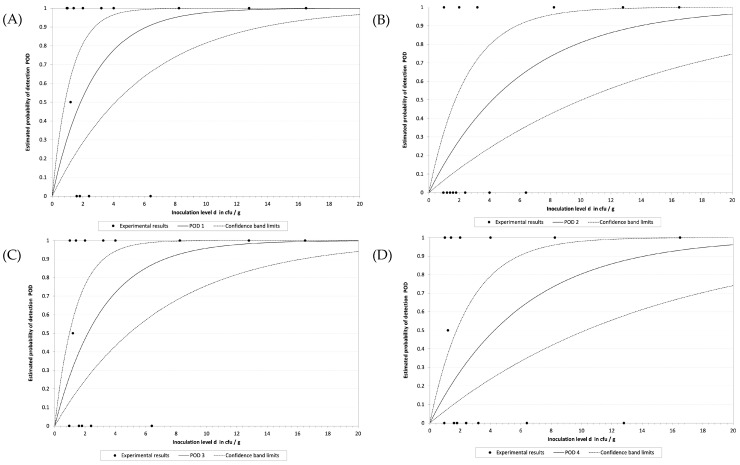
Graphical representation of the calculation of the LOD obtained by the model developed by Wilrich and Wilrich for the TL (**A**), beads (**B**), chelex (**C**) and the GM (**D**) extraction protocols.

To determine the LoD, a total of 30 samples were spiked with different concentrations of *S.* Enteritidis WDCM 00030. Plate counts indicated that the concentration range covered was from 16.5 to 0.96 CFU/ 25 g. Differences in the performance between the DNA extraction protocols were observed. TL showed the lowest LoD with an LoD_50_ of 1.8 CFU/sample, followed by chelex with an LoD_50_ of 2.2 CFU/sample. Magnetic beads and GM presented the highest LoD values with an LoD_50_ of 4.2 and 4.3 CFU/sample, respectively. These results are graphically depicted in Figure 4A–D. Attending to the LoD_50_ values calculated for each DNA extraction protocol, the RLOD values obtained were 1.3, 2.9, 1.5 and 3.0 for TL, beads, chelex and GM, respectively (Table 3).

#### 3.3.2. Fitness-for-Purpose

Once the LoD_50_ was calculated, the samples inoculated above that value were selected for result comparison against the reference method ISO 6579-1:2017. TL and chelex showed only 1 ND, while magnetic beads and GM presented 2 ND. No PDs were detected with either protocol. Considering these results, the SE for TL, beads, chelex and GM was calculated to be 95.7, 92.3, 95.5 and 90.0%, respectively. As no PDs were observed, the SP was 100 % in all cases, resulting in AC values of 96.6, 93.3, 96.3 and 91.7%. Lastly, the k values obtained were 0.90, 0.76, 0.89 and 0.75. All these results are summarized in Table 4.

#### 3.3.3. Interlaboratory Blind Test

The method was also validated with an interlaboratory test. A total of eight samples were received from the organizer. These were reported to be divided into three groups. Two had an initial inoculation level of 10 CFU, another two a level of 57 CFU, and the remaining two samples were not inoculated. There was a total correspondence between the results obtained with LAMP, regardless of the DNA extraction method, and the ISO 6579-1:2017. These results were later confirmed by the organizer of the interlaboratory trial. A total of six samples were positive for *Salmonella* spp., and two samples were negative.

#### 3.3.4. Colorimetric LAMP

**Figure 5:**
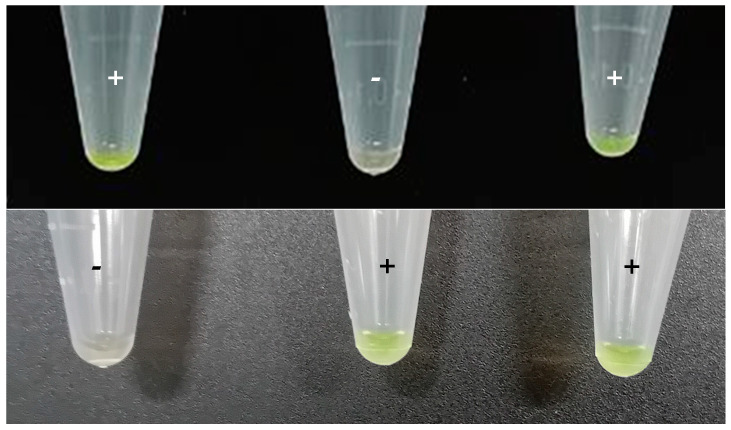
Typical colorimetric results obtained with the *ttr*-LAMP assay. Green tubes are positive reactions, while orange tubes are negative.

Taking into consideration the overall results obtained with the different DNA extraction protocols, it was determined that the TL provided the best results, and so was selected for the colorimetric LAMP proof of concept. All the samples, including the blind interlaboratory ones, analyzed through this alternative protocol returned the same results as those analyzed by real-time *ttr*-LAMP, as shown in Figure 5.

## 4. Discussion

Nowadays, intensive food production systems require rapid responses in order to avoid product release and delivery. This becomes a major challenge when focusing on microbiological determinations due to their reliance on culture-based methods, and the situation is more complex when dealing with the detection of pathogenic bacteria as several days of culture, isolation, identification, and characterization are needed [26]. This is not different for environmental surveillance.

*Salmonella* spp. is one of the most reported human foodborne pathogenic bacteria worldwide. As an example, solely in Europe in 2022, a total of 65208 cases were reported, 5039 cases more compared to 2021, out of which 11,287 needed hospitalization, and resulted in 81 deaths. For the second year in a row since the COVID-19 pandemic, the incidence of salmonellosis has increased [27]. One of the key activities performed to control this pathogen relies on the surveillance of flock breeding beds following the international standard 6579 [19,28].

Molecular methods, mainly PCR/ qPCR-based ones, have already demonstrated suitable for the reduction in analysis turnaround time [29,30]. Unfortunately, they rely on expensive equipment and often result in difficult interpretation. LAMP has the potential to address these issues due to its reliance on single temperature amplification, higher robustness to typical DNA polymerase inhibitors, and compatibility with a wide range of chemicals, which can allow for simple, naked-eye, colorimetric detection, thus opening the door for assay decentralization [13,17,31]. In the present study, a novel method, aligned with ISO 6579 for the detection of *Salmonella* spp., was developed with the idea in mind of paving the way for future application, and implementation in farms. To this end, the *ttr* gene was selected for the design of a new set of LAMP primers. This gene has been previously reported to be suitable in qPCR and LAMP-based methods for the detection of *Salmonella* spp. [32,33,34]. The gene encodes for tetrathionate respiration, which is characteristic of certain genera of the family *Enterobacteriaceae*, including *Salmonella* [35].

Even though studies on the primers targeting the *ttr* gene have been previously published, in-depth characterization was missing; thus, an extensive inclusivity test was performed, including 54 *Salmonella* strains covering 31 serotypes, which were all correctly identified. Regarding the exclusivity, 28 strains of 18 different species were tested, which were all negative. The final step consisted of the determination of the dynamic range, or analytical sensitivity reachable with these primers. This time, two parallel approaches were followed: first, with pure bacterial DNA from a reference strain, and second in a spiked sample. In both cases, the DNA was extracted following four different protocols to determine if any outperformed the others and if they should be included in the final method. These two tests allowed us to better assess the true sensitivity of the assay. When analyzing pure DNA, it was possible to cover a 7-log dynamic range, from ~20–40 ng/µL to 0.2–0.4 pg/µL. The only exception was the chelex protocol with which the dynamic range covered 8 log due to a higher initial concentration (137 ng/ µL). A study performed by Pacheco et al. compared the performance of chelex and silica particles for *Salmonella* DNA extraction from boot swabs, with their results being contrary to those obtained in the present work. It is highly possible that these differences may be explained by variation in the overall extraction and purification process as in our study, a more aggressive lysis protocol was followed prior to the GM application, and no particles/beads of silica were used rather than a silica suspension [36].

When focusing on the performance of the different protocols in spiked samples, no significant differences were observed regarding the DNA concentration recovered. However, when focusing on the purity of the extracts, the best results were obtained with the GM and the beads, as the 260/280 ration was ~1.9, while for TL and chelex, the ration was ~1.7. For pure DNA, a ration of ~1.8 has been reported [37]. A similar scenario was observed when focused on the 260/230 ratio as the GM and the beads had values ~1.3, while the other two protocols were ~0.7. These results were not surprising as the two protocols returning the best results include successive cleaning and purification steps, which allow us to better eliminate contaminating compounds, while the direct TL and the chelex do not; after the heating step, the samples are only centrifuged to pellet food and cellular debris. These results are in line with those reported by Costa-Ribeiro et al. in terms of the DNA concentration and purity ratios obtained with the different protocols in leafy green samples [38].

In consideration of the overall results obtained with the different DNA extraction protocols, it was decided to apply the TL in the final methodology due to its simplicity and lower cost. A study performed by Kim et al. already demonstrated the suitability of the thermal lysis protocol for its combination with molecular methods, even though they raised the potential issue of reaction inhibition due to the absence of DNA purification [39]. Fachmann et al. already demonstrated that, with inhibitor-resistant polymerases, simply applying heat can provide successful sensitive results for the detection of *Salmonella* spp. [40]. It is important to note that the addition of the initial centrifugation step at a low speed was a key step for the effective removal of inhibitory compounds, particularly for the TL and chelex protocols. This approach has also been reported to be suitable for other molecular biology-based methods such as the improvement of host DNA removal for next generation sequencing analyses [41,42].

When proceeding with the validation of the novel methodology, it was determined that the lowest LoD_50_ was reached with TL and the chelex protocols, followed by beads and the GM. Similarly, the best performance parameters were obtained with TL and chelex protocols as it was possible to reach an SE higher than 95% with a k of 0.9 and 0.89, respectively, which were the only protocols that fulfilled the requirements set by NordVal [24]. Even though the beads and the GM protocols provided good results in terms of SE, SP and AC, they failed in the comparison against the reference protocol as the k values obtained were below 0.8, falling into the range of 0.61–0.8, which is interpreted as “substantial agreement”, instead of being in the range of 0.81–1.00, which is interpreted as “almost complete concordance” [43]. In line with this observation was the fact that the RLOD values obtained for TL and chelex were in the range of 0.4 and 2.5 and were considered acceptable, while not deviating significantly from the reference method. The other two protocols obtained values of 2.9 and 3.0, indicating significantly lower performance [44]. It is important to note that all eight interlaboratory blind samples were correctly identified by the *ttr*-LAMP method regardless of the extraction protocol followed, indicating that beads and GM protocols show potential for this application, and with some improvements may reach the desired performance.

Considering the results obtained, the simplicity, and its lower cost, the TL protocol was selected for the colorimetric proof-of-concept. In this sense, the test was successful, opening the door for potential decentralization of the method. Once demonstrated that a simple thermal lysis protocol can be used, and provide sensitive results, the only issue remaining would be to address the enrichment step in a decentralized setup as, due to the selection of LAMP, all other steps of the method may be performed as demonstrated in previous studies [45,46]. In order to reach the needed sensitivity, an enrichment step is still required, as it can be reduced as reported by several authors for different bacterial pathogens [47,48,49,50,51,52], but it cannot be omitted [40].

When focusing on the serotype LAMP assays, it was important to note that cross-amplification occurred with different serotypes. However, these were not considered false positive or unspecific results due to the fact that all the serotypes could be clearly classified based on the melt curve peak obtained, as all five could be clearly differentiated, and no unspecific peaks were obtained. At the present stage, the cross-amplification hinders the applicability of the serotype LAMP as an end-point colorimetric test to be decentralized in a similar way to that of the *ttr*-LAMP. However, this was not considered a major issue as after obtaining a positive result, either on the farm or in the laboratory, the samples must be submitted for bacterial isolation on selective media in order to isolate the bacteria. This process must be performed in a well-equipped laboratory, where a qPCR thermocycler, or other types of real-time fluorescence acquisition devices, is available for serotyping LAMP assays with melt curve confirmation.

## 5. Conclusions

A novel LAMP-based method was developed for the rapid screening of *Salmonella* spp. in broiler flock environmental samples. This novel method demonstrated to be sensitive and highly specific and provided results comparable to those of the ISO 6579-1:2017 reference method, confirmed by the almost complete agreement between both methods with spiked and interlaboratory blinded samples. In addition to this, a colorimetric proof of concept was also performed, and this opens the door for the future development of a methodology suitable for in-farm analysis. Finally, the novel method was coupled with a LAMP-based *Salmonella* spp. serotyping panel for the characterization of the most relevant serotypes, namely *S.* Typhimurium, *S.* Enteritidis, *S*. Infantis, *S*. Virchow and *S.* Hadar. This panel of assays allowed for the identification of these serotypes based on their melt curve profile, reducing the resources needed for the typing of the bacteria, as well as the overall turnaround time needed to perform the detection and characterization of the bacteria.

## Figures and Tables

**Table 1:** Bacterial strains for inclusivity and exclusivity tests.

Strain	Strain	Target					
		*ttr*	STM4497	*safA*	Group_29846	Group_21126	Group_27174
*S.* Typhimurium	WDCM 00031	+	+	-	-	-	-
	C13/22	+	+	-	-	-	-
	C3/21	+	+	-	-	-	-
	C4/20	+	+	-	-	-	-
	C4/22	+	+	-	-	-	-
	C7/21	+	+	-	-	-	-
	C9/20	+	+	-	-	-	-
*S.* Typhimurium monophasi	C15/22	+	+	-	-	-	-
	C9/21	+	+	-	-	-	-
	C9/22	+	+	-	-	-	-
	C7/20	+	+	-	-	-	-
*S.* Enteritidis	C1/20	+	-	+	-	-	-
	C1/21	+	-	+	-	-	-
	C10/20	+	-	+	-	-	-
	C10/21	+	-	+	-	-	-
	C2/22	+	-	+	-	-	-
	C7/22	+	-	+	-	-	-
*S.* Hadar	C11/21	+	-	-	+	-	-
	C2/20	+	-	-	+	-	-
	C3/22	+	-	-	+	-	-
*S.* Infantis	C12/20	+	-	-	-	+	-
	C2/21	+	-	-	-	+	-
*S.* Virchow	C11/22	+	-	-	-	-	+
	C15/20	+	-	-	-	-	+
	C6/21	+	-	-	-	-	+
*S.* Fresno	C13/20	+	-	-	-	-	-
*S.* Lawndale	C6/20	+	-	-	-	-	-
*S.* Abony	C11/20	+	-	-	-	-	-
*S.* Agama	C15/21	+	-	-	-	-	-
*S.* Agbeni	C13/21	+	-	-	-	-	-
*S.* Augustenborg	C12/22	+	-	-	-	-	-
*S.* Berta	C14/21	+	-	-	-	-	-
	C8/22	+	-	-	-	-	-
*S.* Coeln	C8/20	+	-	-	-	-	-
*S.* Dublin	C1/22	+	-	-	-	-	-
	C5/21	+	-	-	-	-	-
*S.* Give	C5/20	+	-	-	-	-	-
*S.* Gloucester	C4/21	+	-	-	-	-	-
*S.* Lagos	C10/22	+	-	-	-	-	-
*S.* Stanley	C12/21	+	-	-	-	-	-
*S.* Stanley	C5/22	+	-	-	-	-	-
*S.* Teddington	C3/20	+	-	-	-	-	-
*S.* Wernigerode	C14/22	+	-	-	-	-	-
	C8/21	+	-	-	-	-	-
*S.* Yoruba	C14/20	+	-	-	-	-	-
*B. cereus*	WDCM 00151	-	NT	NT	NT	NT	NT
*E. faecalis*	20825	-	NT	NT	NT	NT	NT
	CECT 481	-	NT	NT	NT	NT	NT
	WDCM 00009	-	NT	NT	NT	NT	NT
*A. baumannii*	CECT 452	-	NT	NT	NT	NT	NT
*Klebsiella pneumoniae*	CECT 8453	-	NT	NT	NT	NT	NT
*S. uberis*	CECT 994	-	NT	NT	NT	NT	NT
*S. agalactiae*	CECT 183	-	NT	NT	NT	NT	NT
*S. dysgalactiae*	CECT 758	-	NT	NT	NT	NT	NT
*L. monocytogenes*	WDCM 00110	-	NT	NT	NT	NT	NT
	WDCM 00021	-	NT	NT	NT	NT	NT
	L1AM0	-	NT	NT	NT	NT	NT
*L. innocua*	CUP 1375	-	NT	NT	NT	NT	NT
*P. aeruginosa*	WDCM 00024	-	NT	NT	NT	NT	NT
*P. fluorescens*	WDCM 00115	-	NT	NT	NT	NT	NT
*P. fragi*	WDCM 00116	-	NT	NT	NT	NT	NT
*E. coli*	CECT 99	-	NT	NT	NT	NT	NT
	AMC 76	-	NT	NT	NT	NT	NT
	CECT 5947	-	NT	NT	NT	NT	NT
	C179-12	-	NT	NT	NT	NT	NT
*C. difficile*	CECT 531	-	NT	NT	NT	NT	NT
*S. aureus*	WDCM 00034	-	NT	NT	NT	NT	NT
	CECT 54	-	NT	NT	NT	NT	NT
*Y. enterocolitica*	WDCM 00038	-	NT	NT	NT	NT	NT
	WDCM 00039	-	NT	NT	NT	NT	NT
*C. jejuni*	AMC	-	NT	NT	NT	NT	NT
*C. coli*	UM	-	NT	NT	NT	NT	NT
	AMC	-	NT	NT	NT	NT	NT

CECT: Spanish Type Culture Collection. WDCM: World Data Center for Microorganisms. AMC: collection from the Institute of Applied Microbiology, ASMECRUZ. UM: University of Minho. CUP: Catholic University of Porto. NT: Not tested.

**Table 2:** Bacteria, genetic targets and primer sequences.

Bacteria	Target	Primer Name	Sequence (5′→3′)	Reference
*Salmonella* spp.	*ttr*	FIP_ttr	GCA TCA GCC AAC ATA GCG CCA *tttt* CTA CGC CAT CCG TTA TCA CA	[20]
		BIP_ttr	TCA GGT ACA AAC CGT CCC CAA G *tttt* CAT CCG TTC CGC CTG GTA	
		F3_ttr	ACA CTG CTG TTC TGT AGC CT	
		B3_ttr	AGG TGC CGA GAA TAG CCA	
		LF_ttr	CCA GCA GGA CGC GTC TT	
		LB_ttr	CGC GCA ATT TAA CCC TTA CTC G	
*S.* Typhimurium	STM4497	FIP_STM	ACC TGC AGC TCA TTC TGA GCA G *tttt* TCA AAA ACA ACG GCT CCG G	[21]
		BIP_STM	GAA AAG GAC CAC AAG TTC GCG C *tttt* TCA GTG AGC ATG TCG ACG AT	
		F3_STM	AGC CGC ATT AGC GAA GAG	
		B3_STM	GCG GTC AAA TAA CCC ACG T	
*S.* Enteritidis	*safA*	FIP_SEN	AGC CCA CAG TGA GTA TCG TG *tttt* CGC TGC TGG TAG TGC ATG G	[21]
		BIP_SEN	CAG AGG TCA TGG CGC GCA AAT *tttt* GGC ATT GGT ATC AAA GGT GA	
		F3_SEN	GTT GCT AAC ACG ACA CTG GAC	
		B3_SEN	GTG GGA TAT TCT GAG CCC CTA T	
*S.* Infantis	group_21126	FIP_INF	ATA GCC CAC CCC GCA ATT TCG *tttt* GAC TAC ATA CCG TAG CCC CA	Current study
		BIP_INF	CCA GGCG AAT TAG TAT ACG ACC CAT *tttt* TTG AGC CAA GCT TCG AGG A	
		F3_INF	GCA GAT ATC CCA TTA AAA ACT GAG C	
		B3_INF	CGG TAC CAA TAG TAT CCC TAC CT	
		LF_INF	GGG CGC ATC TTC CCA ATG	
		LB_INF	TTT GTG GTT CTG GTA CTG TGC	
*S.* Virchow	group_27174	FIP_VIR	TGG GCC AGC ACA AAT GAA TAC TGT G *tttt* CCA TGA TGG CAA CGG GAT	Current study
		BIP_VIR	TTA GGT GGC ACC CAT CCA GTG *tttt* TAA GGC AGC TCA CAA CGC	
		F3_VIR	TGT ACC TGG TGT TTG ATA TTT CGT	
		B3_VIR	CTG CAA TTG ACC AGT CGG T	
		LF_VIR	TGG ATC TTA AAT AGT CAT CAA ACG A	
		LB_VIR	CTG AAA CTT TTA TTT ATG CTT GGG T	
*S.* Hadar	group_29846	FIP_HAD	GCC GTG ATT TTC TTG ACT AAT TGA T *tttt* CAT GTG GCA ACA TTA GAA CG	Current study
		BIP_HAD	TCT TTG GCG AGA AAA CAG CAA *tttt* TCC TTC ATA AAC GGA ACC G	
		F3_HAD	AGA AGT CCG AGA GGA TGA	
		B3_HAD	ACA GAT TAA GTT CCC TTC CAA	
		LF_HAD	GCA TAC TGA AGC TCT TTT TCT GC	
		LB_HAD	ATT TGC ATT GCT GGC GT	

*tttt* represents a polyT linker between F2 and F1c, and B2 and B1c.

**Table 3:** Samples analyzed.

Concentration Range	N	DNA Extraction Protocol				
		Thermal	Chelex	Beads	GM	ISO
4	1	+	+	+	+	+
3	2	+	+	+	+	+
2	2	+	+	+	+	+
1	6	+	+	+	5	+
<1	16	8	7	4	6	8
NS	2	-	-	-	-	-
HPI	2	+	+	+	+	+
LPI	2	+	+	+	+	+
NI	2	-	-	-	-	-

Concentration range values expressed as “log CFU/ sample”. “N”: number of samples. A “+” or “-” denotes that all the samples were either positive or negative; alternatively, if a number is provided, it indicates the number of positive samples. “ISO” refers to the reference method ISO 6579-1:2017. “NS”: not spiked. “HPI”: high positive interlaboratory samples (57 CFU). “LPI”: low positive interlaboratory samples (10 CFU). “NI”: negative interlaboratory samples.

**Table 4:** Evaluation summary.

DNA Extraction Protocol	LoD_50_	RLOD	N	PA	NA	PD	ND	SE	SP	AC	k
TL	1.8	1.3	29	22	6	0	1	95.7	100.0	96.6	0.90
Beads	4.2	2.9	30	24	4	0	2	92.3	100.0	93.3	0.76
Chelex	2.2	1.5	27	21	5	0	1	95.5	100.0	96.3	0.89
GM	4.3	3.0	24	18	4	0	2	90.0	100.0	91.7	0.75

“TL”: thermal lysis. “GM”: thermal lysis with glass milk purification. LoD_50_: Limit of Detection 50%. RLOD: Relative Limit of Detection, with respect to ISO 6579-1:2017. PA, NA, PD and ND are “Positive Agreement”, “Negative Agreement”, “Positive Deviation” and “Negative Deviation”, respectively. SE, SP and AC are the relative sensitivity, specificity and accuracy, respectively, and the “k” is Cohen’s kappa value.

## Data Availability

The original contributions presented in the study are included in the article, further inquiries can be directed to the corresponding authors.

## References

[1] Mottet A., Tempio G. (2017). Global Poultry Production: Current State and Future Outlook and Challenges. Worlds Poult. Sci. J..

[2] European Commission (2003). Regulation (EC) No 2160/2003 of the European Parliament and of the Council of 17 November 2003 on the Control of *Salmonella* and Other Specified Food-Borne Zoonotic Agents. Off. J..

[3] (2023). MAPA; Ministerio de Agricultura, P. y A. PROGRAMA NACIONAL DE CONTROL DE DETERMINADOS DE *Salmonella* EN GALLINAS PONEDO DE LA ESPECI *Gallus gallus*.

[4] Talorico A.A., Bailey M.A., Munoz L.R., Chasteen K.S., Pal A., Krehling J.T., Bourassa D.V., Buhr R.J., Macklin K.S. (2021). The Use of Roller Swabs for *Salmonella* Detection in Poultry Litter. J. Appl. Poult. Res..

[5] Mueller-Doblies D., Sayers A.R., Carrique-Mas J.J., Davies R.H. (2009). Comparison of Sampling Methods to Detect *Salmonella* Infection of Turkey Flocks. J. Appl. Microbiol..

[6] European Commission Commission Regulation (EU) No 200/2010.Implementing Regulation (EC) No 2160/2003 of the European Parliament and of the Council as Regards a Union Target for the Reduction of the Prevalence of *Salmonella* Serotypes in Adult Breeding Flocks of Gallus Gallus. https://eur-lex.europa.eu/eli/reg/2010/200/2019-03-10.

[7] Commission Regulation (EC) No 2073/2005 Microbiological Criteria for Foodstuffs 2005, 2073/2005. https://eur-lex.europa.eu/eli/reg/2005/2073/oj.

[8] Tomás D., Rodrigo A., Hernández M., Ferrús M.A. (2009). Validation of Real-Time PCR and Enzyme-Linked Fluorescent Assay-Based Methods for Detection of *Salmonella* spp. in Chicken Feces Samples. Food Anal. Methods.

[9] Ding Y., Huang C., Zhang Y., Wang J., Wang X. (2023). Magnetic Microbead Enzyme-Linked Immunoassay Based on Phage Encoded Protein RBP 41-Mediated for Rapid and Sensitive Detection of *Salmonella* in Food Matrices. Food Res. Int..

[10] Hice S.A., Clark K.D., Anderson J.L., Brehm-Stecher B.F. (2018). Capture, Concentration and Detection of *Salmonella* in Foods Using Magnetic Ionic Liquids and Recombinase Polymerase Amplification. Anal. Chem..

[11] Carra E., Galletti G., Carpana E., Bergamini F., Loglio G., Bosi F., Palminteri S., Bassi S. (2022). A Probe-Based QPCR Method, Targeting 16S rRNA Gene, for the Quantification of *Paenibacillus larvae* Spores in Powdered Sugar Samples. Appl. Sci..

[12] Zhou S., Lou E.G., Schedler J., Ensor K.B., Hopkins L., Stadler L.B. (2024). Comparative Analysis of Culture- and ddPCR-Based Wastewater Surveillance for Carbapenem-Resistant Bacteria. Environ. Sci. Water Res. Technol..

[13] Soroka M., Wasowicz B., Rymaszewska A. (2021). Loop-Mediated Isothermal Amplification (LAMP): The Better Sibling of PCR?. Cells.

[14] Nguyen H.Q., Bui H.K., Phan V.M., Seo T.S. (2022). An Internet of Things-Based Point-of-Care Device for Direct Reverse-Transcription-Loop Mediated Isothermal Amplification to Identify SARS-CoV-2. Biosens. Bioelectron..

[15] Piepenburg O., Williams C.H., Stemple D.L., Armes N.A. (2006). DNA Detection Using Recombination Proteins. PLoS Biol..

[16] Notomi T., Okayama H., Masubuchi H., Yonekawa T., Watanabe K., Amino N., Hase T. (2000). Loop-Mediated Isothermal Amplification of DNA. Nucleic Acids Res..

[17] Garrido-Maestu A., Prado M. (2022). Naked-Eye Detection Strategies Coupled with Isothermal Nucleic Acid Amplification Techniques for the Detection of Human Pathogens. Compr. Rev. Food Sci. Food Saf..

[18] Selva Sharma A., Lee N.Y. (2024). Advancements in Visualizing Loop-Mediated Isothermal Amplification (LAMP) Reactions: A Comprehensive Review of Colorimetric and Fluorometric Detection Strategies for Precise Diagnosis of Infectious Diseases. Coord. Chem. Rev..

[19] (2017). Microbiology of the Food Chain—Horizontal Method for the Detection, Enumeration and Serotyping of Salmonella—Part 1: Detection of Salmonella spp.

[20] Costa-Ribeiro A., Lamas A., Garrido-Maestu A. (2024). Evaluating Commercial Loop-Mediated Isothermal Amplification Master Mixes for Enhanced Detection of Foodborne Pathogens. Foods.

[21] Garrido-Maestu A., Fuciños P., Azinheiro S., Carvalho J., Prado M. (2017). Systematic Loop-Mediated Isothermal Amplification Assays for Rapid Detection and Characterization of *Salmonella* spp., Enteritidis and Typhimurium in Food Samples. Food Control.

[22] Wilrich C., Wilrich P.T. (2009). Estimation of the Pod Function and the LOD of a Qualitative Microbiological Measurement Method. J. AOAC Int..

[23] Mărgăritescu I., Wilrich P.-T. (2013). Determination of the Relative Level of Detection of a Qualitative Microbiological Measurement Method with Respect to a Reference Measurement Method. J. AOAC Int..

[24] (2016). NordVal International Protocol for the Validation of Microbiological Alternative (Proprietary) Methods Against a Reference Method. https://www.semanticscholar.org/paper/NordVal-International-Protocol-for-the-validation-a/3e4849b0dbd841d01af148feb66d21c19fb596d7.

[25] Anderson A., Pietsch K., Zucker R., Mayr A., Müller-Hohe E., Messelhäusser U., Sing A., Busch U., Huber I., Muller-Hohe E. (2011). Validation of a Duplex Real-Time PCR for the Detection of *Salmonella* spp. in Different Food Products. Food Anal. Methods.

[26] Valderrama W.B., Dudley E.G., Doores S., Cutter C.N. (2016). Commercially Available Rapid Methods for Detection of Selected Food-Borne Pathogens. Crit. Rev. Food Sci. Nutr..

[27] Report S. (2023). The European Union One Health 2022 Zoonoses Report. EFSA J..

[28] (2020). Microbiology of the Food Chain—Horizontal Method for the Detection, Enumeration and Serotyping of *Salmonella*—Part 1: Detection of *Salmonella* spp.—Amendment 1: Broader Range of Incubation Temperatures, Amendment to the Stat 2020.

[29] Ferone M., Gowen A., Fanning S., Scannell A.G.M. (2020). Microbial Detection and Identification Methods: Bench Top Assays to Omics Approaches. Compr. Rev. Food Sci. Food Saf..

[30] Chapela M.-J., Garrido-Maestu A., Cabado A.G. (2015). Detection of Foodborne Pathogens by QPCR: A Practical Approach for Food Industry Applications. Cogent Food Agric..

[31] Niessen L., Luo J., Denschlag C., Vogel R.F. (2013). The Application of Loop-Mediated Isothermal Amplification (LAMP) in Food Testing for Bacterial Pathogens and Fungal Contaminants. Food Microbiol..

[32] Kreitlow A., Becker A., Schotte U., Malorny B., Plötz M., Abdulmawjood A. (2021). Evaluation of Different Target Genes for the Detection of *Salmonella* sp. by Loop-Mediated Isothermal Amplification. Lett. Appl. Microbiol..

[33] González-Escalona N., Brown E.W., Zhang G. (2012). Development and Evaluation of a Multiplex Real-Time PCR (qPCR) Assay Targeting ttrRSBCA Locus and *invA* Gene for Accurate Detection of *Salmonella* spp. in Fresh Produce and Eggs. Food Res. Int..

[34] Malorny B., Paccassoni E., Fach P., Bunge C., Martin A., Helmuth R. (2004). Diagnostic Real-Time PCR for Detection of *Salmonella* in Food. Appl. Environ. Microbiol..

[35] Barrett E.L., Clark M.A. (1987). Tetrathionate Reduction and Production of Hydrogen Sulfide from Thiosulfate. Microbiol. Rev..

[36] Pacheco J.I.M., dos Anjos K.B.A., Silva I.V., Okar R.G., Rodrigues S.M.B.D., Francabandiera A.I., Rodriguez M.C. (2023). Comparison of Two Affordable DNA Extraction Methods for Molecular Detection of Salmonella Isolates from Broiler Farm’s Boot Swabs. Res. Soc. Dev..

[37] Geuther R. (2007). 260/280 and 260/230 Ratios Introduction. Z. Allg. Mikrobiol..

[38] Costa-Ribeiro A., Lamas A., Mora A., Prado M., Garrido-Maestu A. (2024). Moving towards On-Site Detection of Shiga Toxin-Producing *Escherichia coli* in Ready-to-Eat Leafy Greens. Curr. Res. Food Sci..

[39] Kim J.H., Jung S., Oh S.W. (2020). Combination of Bacteria Concentration and DNA Concentration for Rapid Detection of *E. coli* O157:H7, *L. monocytogenes*, and *S*. Typhimurium without Microbial Enrichment. LWT.

[40] Fachmann M.S.R., Josefsen M.H., Hoorfar J., Nielsen M.T., Löfström C. (2015). Cost-Effective Optimization of Real-Time PCR-Based Detection of *Campylobacter* and *Salmonella* with Inhibitor Tolerant DNA Polymerases. J. Appl. Microbiol..

[41] Ong C.T., Boe-Hansen G., Ross E.M., Blackall P.J., Turni C., Hayes B.J., Tabor A.E. (2022). Evaluation of Host Depletion and Extraction Methods for Shotgun Metagenomic Analysis of Bovine Vaginal Samples. Microbiol. Spectr..

[42] Garrido-Maestu A., Lamas A., Fornés D.T., Rodríguez M.P., Bridier A. (2025). The Use of Multiplex Real-Time PCR for the Simultaneous Detection of Foodborne Bacterial Pathogens. Foodborne Bacterial Pathogens: Methods and Protocols.

[43] Altman D.G., Hall C. (1991). Practical Statistics for Medical Research.

[44] Zheng J., Reed E., Maounounen-Laasri A., Deng X., Wang S.S., Ramachandran P., Ferreira C., Bell R., Brown E.W., Hammack T.S. (2024). Evaluation of Universal Preenrichment Broth and Comparison of Rapid Molecular Methods for the Detection of *Salmonella* from Spent Sprout Irrigation Water (SSIW). Int. J. Food Microbiol..

[45] Fujiyoshi S., Yarimizu K., Miyashita Y., Rilling J., Acuña J.J., Ueki S., Gajardo G., Espinoza-González O., Guzmán L., Jorquera M.A. (2021). Suitcase Lab: New, Portable, and Deployable Equipment for Rapid Detection of Specific Harmful Algae in Chilean Coastal Waters. Environ. Sci. Pollut. Res..

[46] Higgins O., Chueiri A., Connor L.O., Lahiff S., Burke L., Morris D., Pfeifer M. (2023). Portable Differential Detection of CTX-M ESBL Gene Variants, *bla*_CTX-M-1_ and *bla*_CTX-M-15_, from Escherichia coli Isolates and Animal Fecal Samples Using Loop-Primer Endonuclease Cleavage Loop-Mediated Isothermal Amplification. Microbiol. Spectr..

[47] Fachmann M.S.R., Löfström C., Hoorfar J., Hansen F., Christensen J., Mansdal S., Josefsen M.H.M.H., Mandal S., Josefsen M.H.M.H. (2017). Detection of *Salmonella* Enterica in Meat in Less than 5 Hours by a Low-Cost and Noncomplex Sample Preparation Method. Appl. Environ. Microbiol..

[48] Costa-Ribeiro A., Azinheiro S., Fernandes S.P.S., Lamas A., Prado M., Salonen L.M., Garrido-Maestu A. (2023). Evaluation of Covalent Organic Frameworks for the Low-Cost, Rapid Detection of Shiga Toxin-Producing *Escherichia coli* in Ready-to-Eat Salads. Anal. Chim. Acta.

[49] Lamas A., Santos S.B., Prado M., Garrido-Maestu A. (2023). Phage Amplification Coupled with Loop-Mediated Isothermal Amplification (PA-LAMP) for Same-Day Detection of Viable *Salmonella* Enteritidis in Raw Poultry Meat. Food Microbiol..

[50] Rossmanith P., Suss B., Wagner M., Hein I. (2007). Development of Matrix Lysis for Concentration of Gram Positive Bacteria from Food and Blood. J. Microbiol. Methods.

[51] Mann E., Pommer K., Mester P., Wagner M., Rossmanith P. (2014). Quantification of Gram-Positive Bacteria: Adaptation and Evaluation of a Preparation Strategy Using High Amounts of Clinical Tissue. BMC Vet. Res..

[52] Mester P., Wagner M., Rossmanith P. (2018). Molecular Enrichment for Qualitative Molecular Pathogen Detection in Food. Food Anal. Methods.

